# A brief guide to analyzing expression quantitative trait loci

**DOI:** 10.1016/j.mocell.2024.100139

**Published:** 2024-10-22

**Authors:** Byung Su Ko, Sung Bae Lee, Tae-Kyung Kim

**Affiliations:** 1 Department of Brain Sciences, DGIST, Daegu 42988, Republic of Korea; 2 Department of Life Sciences, Pohang University of Science and Technology (POSTECH), Pohang 37673, Republic of Korea; 3 Institute for Convergence Research and Education in Advanced Technology, Yonsei University, Seoul 03722, Republic of Korea

## Abstract

Molecular quantitative trait locus (molQTL) mapping has emerged as an important approach for elucidating the functional consequences of genetic variants and unraveling the causal mechanisms underlying diseases or complex traits. However, the variety of analysis tools and sophisticated methodologies available for molQTL studies can be overwhelming for researchers with limited computational expertise. Here, we provide a brief guideline with a curated list of methods and software tools for analyzing expression quantitative trait loci, the most widely studied type of molQTL.

## INTRODUCTION

In recent decades, genome-wide association studies (GWAS) have advanced our understanding of the genetic basis of diseases and complex traits by identifying causal variants present in human populations (Buniello et al., 2019, Wang et al., 2022, Visscher et al., 2017). To decipher the underlying mechanisms and discover potential therapeutic targets, there is a growing need to interpret the functional relevance of genetic variants (Cano-Gamez and Trynka, 2020). With the rapid advancements in high-throughput sequencing technologies, an increasing number of studies have adopted integrative approaches combining genetic information with various molecular phenotypes, such as gene expression, splicing, protein abundance, and chromatin modification/accessibility. These integrative strategies have paved the way for molecular quantitative trait loci (molQTL) mapping (Aguet et al., 2023), a powerful statistical framework that identifies genetic loci associated with quantitative variations in molecular phenotypes, thereby providing insights into the functional consequences of genetic variants.

**Fig. 1:**
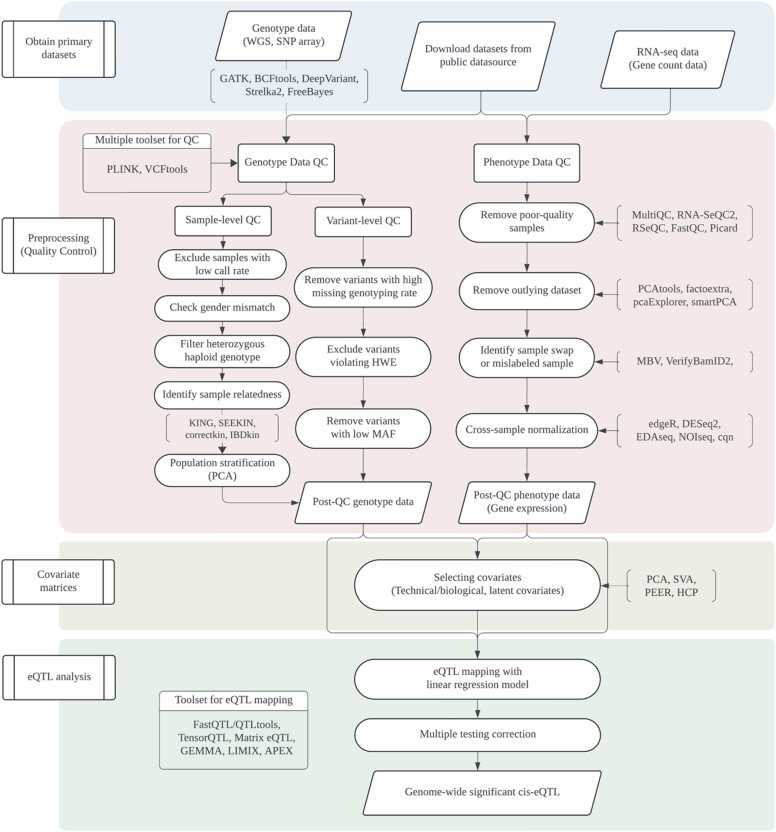
Framework diagram of eQTL mapping. A schematic diagram of the eQTL mapping process with commonly used tools for each step. eQTL, expression quantitative trait loci; HWE, Hardy-Weinberg Equilibrium; QC, quality control; MAF, minor allele frequency; WGS, whole-genome sequencing.

Expression quantitative trait loci (eQTL) mapping determines the regulatory effects of genetic variants on gene expression levels, which can provide insights into disease mechanisms. Large-scale consortia, such as the eQTL Catalogue (Kerimov et al., 2021, Kerimov et al., 2023), the Genotype-Tissue Expression (GTEx) project (GTex Consortium, 2020), and the eQTLGen consortium (Vosa et al., 2021) offer catalogs of eQTL summaries and annotations in diverse human tissues. Given the population scale of genome-wide studies, robust eQTL analysis typically requires genetic data from hundreds of individuals to achieve sufficient statistical power (Huang et al., 2018). A wide range of computational tools and methodologies have been developed and integrated into bioinformatics pipelines to facilitate the analysis of large-scale genetic and phenotypic datasets (Kel et al., 2016, Kerimov et al., 2021, Lee et al., 2024, Wang et al., 2021, Yoo et al., 2021). Although eQTL mapping protocols and summary statistics are publicly accessible, researchers with limited computational expertise may encounter challenges in orchestrating computational workflows and processing large-scale datasets. Here, we provide a curated resource for eQTL mapping analysis to assist experimental biologists (Fig. 1 and Supplementary Table).

## MAIN BODY

### Overview of eQTL Mapping

eQTL mapping requires 2 types of datasets: genotype data and gene expression data. Before eQTL mapping, quality control (QC) of both datasets should be conducted to identify and remove problematic or outlying samples, preventing a loss of power in subsequent eQTL analysis. Using QC-processed datasets, eQTL mapping identifies genetic variants that significantly affect the expression levels of putative target genes, providing insights into the regulatory networks of gene expression. It should be noted that the statistical power of eQTL studies is highly dependent on sample size. Small sample sizes can lead to false positives or false negatives, thereby reducing the reliability of the results (Huang et al., 2018). To enhance the robustness of eQTL findings, researchers should aim for larger sample sizes or consider conducting meta-analyses that combine data from multiple studies (Sieberts et al., 2020).

### Genotype Data

Genome-wide genotype data, obtained from whole-genome sequencing and/or single nucleotide polymorphism arrays combined with genotype imputation, provide comprehensive coverage of genetic variations across the genome and enhance the power to identify causal variants. Variant calling tools such as the Genome Analysis Toolkit (GATK) (McKenna et al., 2010), BCFtools (Li, 2011), DeepVariant (Poplin et al., 2018), Strelka2 (Kim et al., 2018), and FreeBayes (Garrison and Marth, 2012) are employed to detect variants from sequencing and microarray data. Among these tools, GATK, a widely adopted suite of tools developed by the Broad Institute, analyzes high-throughput sequencing data to discover genetic variants and integrates information on variants into VCF (variant call format) files (Danecek et al., 2011). VCF files can also be obtained from public repositories such as dbSNP (https://www.ncbi.nlm.nih.gov/snp/), the 1000 Genomes Project (Genomes Project et al., 2015), gnomAD (https://gnomad.broadinstitute.org/), EVA (https://www.ebi.ac.uk/eva/), or UK Biobank, (Bycroft et al., 2018), or from individual research publications.

QC of genotype data is an indispensable step to ensure the reliability and accuracy of eQTL analysis. Several QC tools such as PLINK (Chang et al., 2015, Marees et al., 2018, Purcell et al., 2007) and VCFtools (Danecek et al., 2011) offer a range of functionalities (eg, data formatting, filtering, and statistical analyses) to perform the overall genotype QC process. In this resource, we organized the multiple steps of genotype QC into 2 levels: sample-level QC and variant-level QC.

#### Sample-level QC

When combining genotype data from heterogeneous sources, missing genotypes per sample can be a common issue. VCFtools (*--missing-indv*) or PLINK (*--mind*) calculates the missing rate of genotypes for each sample, allowing the exclusion of low-quality samples with excessive missing genotypes.

Gender mismatches can be detected by examining the homozygosity rate of genetic variants on the X chromosome by using PLINK (*--check-sex*) (Zhao et al., 2018). For instance, males have a higher expected homozygosity rate of 1, while females have a lower rate. Comparing reported sex information with the observed homozygosity rates identifies gender discrepancies, and the corresponding samples should be removed. Even after excluding gender-mismatched samples, heterozygous haploid genotypes of variants on the X chromosome may persist due to errors in genotype calling or sequencing. To maintain data integrity, these genotypes should also be treated as missing and removed by using PLINK (*--set-hh-missing*).

To reduce the false-positive rates in eQTL mapping analysis, the relatedness between each pair of samples should be assessed. The kinship coefficient, a common measure of relatedness, is defined as the probability that a pair of randomly sampled homologous alleles derived from 2 individuals are identical by descent (Speed and Balding, 2015, Thompson, 1975). One issue in the relatedness estimation is that the presence of strong linkage disequilibrium (LD) among detected variants can lead to overestimation of relatedness. Hence, LD pruning is often recommended to improve the estimation accuracy of relatedness by reducing the number of redundant variants in strong LD. PLINK (*--indep-pairwise*) command is a widely used tool for LD pruning, effectively removing variants that are highly correlated with each other. The algorithm calculates pairwise LD (*r*^2^) between all variants within a specified window, identifying variants in strong LD and subsequently removing 1 variant that exceeds a certain LD threshold. Following LD pruning, researchers can employ specialized tools such as KING (Manichaikul et al., 2010), SEEKIN (Dou et al., 2017), correctkin (Nyerki et al., 2023), and IBDkin (Zhou et al., 2020) to identify related individuals in each experiment by setting a certain threshold for expected kinship coefficients. Then, researchers can either remove 1 individual from each related pair or adjust for relatedness in the eQTL analysis using a linear mixed model, which incorporates kinship coefficients into kinship matrices to account for population structure and confounding effects (Hoffman, 2013, Lee, 2018, Pala et al., 2017).

Population stratification is another crucial factor to consider in eQTL mapping. It refers to the existence of systematic differences in allele frequencies between subpopulations, which can be attributed to variations in ancestry or geographic origin (Yang et al., 2014). These differences can introduce confounding effects, potentially leading to false-positive or false-negative associations between genetic variants and gene expression levels. To mitigate this issue, principal component analysis (PCA) has been widely adopted to identify population structure and potential relatedness (Price et al., 2006). Principal components (PCs) defined from LD-pruned datasets can be used to identify and remove outlying samples that deviate from the primary ancestral clusters. These PCs, derived from genotype data, can then be incorporated as covariates in the eQTL model to adjust for population structures (see the section on Selecting Covariates).

#### Variant-level QC

Since the differences in read depth of genes across experiments could potentially lead to missing genotypes, variants with a high missing genotyping rate should be removed to prevent false-positive signals in subsequent analyses. Missingness can be identified by using PLINK (*--geno*) or VCFtools (*--max-missing*) options.

To identify potential genotyping errors and population stratifications, researchers should confirm whether genetic variants violate the principle of Hardy-Weinberg Equilibrium (HWE), which assumes constant genotype and allele frequencies across generations in a large population without natural selection, newly occurred mutations, or gene migration (Wang et al., 2021, Wigginton et al., 2005). The Chi-squared test is commonly used to assess HWE violations. This test compares the observed genotype frequencies with the expected frequencies under the assumption of HWE (Rohlfs and Weir, 2008) and generates a *P*-value that indicates the significance of the deviation. In practice, an HWE *P*-value threshold of 10^−6^ is commonly used to filter out variants that significantly deviate from HWE, ensuring high-quality variants for downstream analyses.

Variants with a minor allele frequency (MAF) below a certain threshold are often removed in eQTL mapping studies to reduce computational burden and false-positive associations. These variants have limited statistical power to detect significant associations with gene expression. Therefore, removing low MAF variants allows subsequent analyses to prioritize variants with sufficient statistical power, enhancing the overall robustness and reliability of the eQTL results. Several tools, including PLINK (*--maf*) and VCFtools (*--freq*), can be used to detect and filter out MAF variants below the set threshold, which depends on sample size and study design (Hong and Park, 2012). For instance, a higher MAF threshold may be appropriate in studies with smaller sample sizes to ensure sufficient statistical power.

### Phenotype Data

Publicly available RNA-seq datasets may be provided in various formats depending on the processing steps (eg, raw read data, read-aligned data, feature-counted data, or standardized data) (Sanchis et al., 2021). RNA-seq data compiled in different formats must be integrated into a single format for subsequent analysis. As a basic QC measure for RNA-seq datasets, low-quality samples (eg, those with poor sequencing quality or a low percentage of mapped reads) should be identified and excluded. Additionally, genes that exhibit expression levels below a defined threshold across samples should be filtered out to reduce noise. These QC procedures can be performed by using the following software tools: MultiQC (Ewels et al., 2016), RNA-SeQC2 (Graubert et al., 2021), RSeQC (Wang et al., 2012), FastQC (Andrews, 2010), and Picard (https://github.com/broadinstitute/picard).

After data integration, it is essential to identify and remove samples that exhibit atypical gene expression profiles. These outliers may arise from technical issues such as sample contamination or failures in RNA-seq library preparation. PCA on the RNA-seq data, using the first 2 components to capture major variations, can detect potential outliers by using tools such as PCAtools, factoextra, pcaExplorer, and smartPCA from the EIGENSOFT package (https://github.com/DReichLab/EIG).

To ensure the integrity of RNA-seq datasets, researchers should identify and correct sample swaps, mislabeled samples, or cross-contamination between RNA-seq samples by using tools such as Match Bam to VCF (Fort et al., 2017) and VerifyBamID2 (Zhang et al., 2020). In addition, gender-mismatched samples can be identified by measuring the gene expression levels of gender-specific genes, such as the *RPSY41* or *XIST* gene.

RNA-seq data normalization is a crucial step enabling the comparison of gene expression levels across samples. While intrasample normalization, such as CPM/FPKM/RPKM/TPM, normalizes gene expression levels within individual samples, it is not well-suited for comparing expression levels across samples and experiments. To address this issue, various software packages (edgeR [Robinson et al., 2010], DESeq2 [Love et al., 2014], NOIseq [Tarazona et al., 2015], cqn [Hansen et al., 2012], and EDAseq [Risso et al., 2011]) provide tools for cross-sample normalization of RNA-seq data, such as Trimmed Mean of M values (Robinson and Oshlack, 2010), Relative Log Expression (Abbas-Aghababazadeh et al., 2018), Quantile normalization (Bolstad et al., 2003, Cloonan et al., 2008), and Median Ratio Normalization (Dillies et al., 2013). These methods remove systematic biases that can arise from technical variations, such as library preparation or sequencing platforms. Following cross-sample normalization, gene expression data should be transformed by the inverse normal transformation method, which converts the data to follow a normal distribution and aligns it with the assumptions of regression models in subsequent analyses. The inverse normal transformation enhances the comparability of gene expression levels across samples by reducing the impact of outliers.

### Selecting Covariates

Adjustment of covariates accounts for unwanted variations introduced by confounding factors, enhancing the power to detect true associations between genetic variants and gene expression levels. Both technical (eg, batch effects, RNA-seq features such as read length, paired/single-end sequencing, library size, sequencing platform) and biological covariates (eg, age, sex, tissue type) can be regressed out using linear regression models. However, including too many covariates can lead to overfitting, which reduces the statistical power of detection and produces unreliable estimates of eQTL effects. Therefore, researchers should carefully consider and prioritize the most relevant covariates for inclusion in the regression models.

In addition to known covariates, latent covariates that are not directly observable or measurable can be inferred from the patterns present in each dataset. PCs derived from both genotype and phenotype data can capture latent sources of variations. The number of PCs is often determined by identifying the elbow point of the scree plot, which depicts the proportion of variance in each PC, to improve the accuracy of the analysis (Zhou et al., 2022). In addition to PC analysis, statistical methods such as surrogate variable analysis (Leek and Storey, 2007), probabilistic estimation of expression residuals (Stegle et al., 2012), or hidden covariates with prior (Mostafavi et al., 2013) can also be employed to capture latent covariates from gene expression data and to control for confounding factors. The optimal number of these covariates to be included in the analysis can be determined by maximizing the number of detected eQTLs while minimizing the risk of overfitting.

### eQTL Mapping

The primary goal of eQTL mapping is to identify genetic variants that influence gene expression levels and determine whether these associations exhibit genome-wide significance. In eQTL analysis, normalized gene expression values, covariate matrices, genotypes, and regression models are used to identify statistically significant associations between variants and phenotypes, thereby discovering QTLs in *cis* (proximal) and *trans* (distal) regions (de Klein et al., 2023, GTEx Consortium, Laboratory D. A., Coordinating Center -Analysis Working G., 2017, Kerimov et al., 2021). *Cis*-regulatory variants affect gene expression through regulatory elements located near the target genes, typically within a 100 kb to 1 Mb window from the transcription start site. In contrast, *trans*-regulatory variants influence gene expression from a different chromosome or at least 5 Mb away from the target genes. This resource focuses on mapping *cis*-eQTLs, which are more commonly analyzed due to their larger effect sizes and stronger effects of variants on gene expression than *trans*-eQTLs (Liu et al., 2019, Pierce et al., 2014, Wang et al., 2024).

eQTL analysis involves association tests between genetic variants and gene expression levels across the genome. Nominal *P*-values of correlation for each variant-gene pair are calculated using significance tests based on the null hypothesis of no association between the variant and gene expression. However, selecting an appropriate genome-wide significance threshold for eQTL mapping is challenging due to a large number of association tests conducted with every possible variant-gene pair across the entire genome. Therefore, multiple testing correction is applied to control the false discovery rate (FDR), ensuring the genome-wide significance of the identified associations. Several multiple testing correction methods, such as Bonferroni correction, FDR correction, or permutation-based methods, can be applied to adjust the *P*-values or significance threshold. Selecting the appropriate multiple-testing correction method depends on the study design, number of tests, effect sizes of the eQTLs, and the specific research objectives. For studies aiming to uncover a comprehensive list of potential eQTLs, a less stringent method like FDR might be more suitable. On the other hand, studies requiring high confidence in the identified eQTLs might prefer a more stringent correction method, such as the Bonferroni correction. However, the conservative nature of this method can increase the risk of false negatives, potentially missing true associations with smaller effect sizes. Researchers should carefully select a correction method that balances minimizing false positives with the ability to detect true associations, considering the objectives of their study.

To efficiently perform a large number of association tests for eQTL mapping and subsequent multiple testing correction, several software packages have been developed, including Matrix eQTL (Shabalin, 2012), FastQTL/QTLtools (Ongen et al., 2016, Delaneau et al., 2017), and TensorQTL (Taylor-Weiner et al., 2019). While Matrix eQTL provides a basic framework for eQTL mapping, FastQTL/QTLtools improves its computational efficiency. These tools model the null distribution of the association test statistics using a beta distribution. This approach enables the estimation of adjusted *P*-values with fewer permutations, thereby mitigating the computational burden. TensorQTL utilizes the computational power of TensorFlow, which employs tensor operation and graphics processing unit acceleration to speed up the computation of association tests. GEMMA (Zhou and Stephens, 2012), LIMIX (Casale et al., 2015), and APEX (Corbin et al., 2020) are specifically designed for eQTL mapping using linear mixed models as a regression model.

### Translating eQTL Findings Into Meaningful Biological Insights

While eQTL mapping provides a robust statistical framework for identifying associations between genetic variants and gene expression, the true value of these studies lies in uncovering the biological mechanisms driving these associations and their relevance to diseases. To translate statistical associations into biological insights, it is crucial to identify causal variants and understand their regulatory mechanisms. Fine-mapping methods play a critical role in this process by refining the list of candidate causal variants within an eQTL region. Tools such as CAVIAR (Hormozdiari et al., 2014), DAP-G (Wen et al., 2016), FINEMAP (Benner et al., 2016), and SuSiE (Wang et al., 2020) employ statistical models to estimate the probability that a given variant affects gene expression, producing credible sets of variants at a specified probability threshold.

Once potential causal variants are prioritized, integration of functional genomic annotations becomes essential for biological interpretation. These annotations include overlaps with regulatory elements (eg, enhancers, promoters), chromatin accessibility data (eg, ATAC-seq or DNase-seq data), and histone modifications. Databases such as ENCODE (Consortium, 2012) provide these annotations, enabling researchers to contextualize eQTL variants within the regulatory landscape. Given the highly context-dependent nature of gene expression, validating the tissue specificity of identified eQTLs is crucial. The GTEx project exemplifies this approach by conducting eQTL mapping across multiple human tissues and integrating tissue-specific functional annotations, providing a comparative framework for eQTL analysis (GTEx Consortium, 2020). This approach allows researchers to determine whether identified eQTLs are tissue-specific or shared across multiple tissues, thereby linking genetic variants to relevant biological functions and potential disease associations.

Colocalization analysis enhances the biological interpretation of eQTLs by determining whether the same genetic variant underlies both an eQTL signal, indicating the association between a variant and gene expression levels, and a GWAS signal, which links genetic variants to complex traits or diseases. By using tools like coloc (Giambartolomei et al., 2014) and eCAVIAR (Hormozdiari et al., 2016), this approach helps to uncover causal relationships between genetic variants and disease risk, allowing researchers to pinpoint specific genes or regulatory elements that may contribute to disease mechanisms. For instance, a study on cerebral cortical development identified eQTLs that overlap with GWAS loci for neuropsychiatric disorders, such as schizophrenia and autism, providing insights into disease mechanisms and potential therapeutic targets (Werling et al., 2020). Moreover, integrating eQTL data with other omics layers offers a more comprehensive understanding of how genetic variants influence cellular pathways and biological processes. In the context of cancer, a recent study combined multiple layers of omics data, including genomic, transcriptomic, proteomic, and phosphoproteomic information, to reveal key signaling pathways and protein networks involved in tumor progression (Chen et al., 2020). By leveraging these multiomics data, researchers can gain deeper insights into the pathological mechanisms underlying various diseases, thereby enhancing the precision and effectiveness of targeted therapies.

While computational methods provide strong evidence for eQTL associations, experimental validation is crucial for establishing causality. Techniques such as massively parallel reporter assays (Tewhey et al., 2016) and Clustered Regularly Interspaced Short Palindromic Repeats-based genome editing (Gasperini et al., 2019) complement computational methods by providing functional evidence of the causal relationship between genetic variants and gene expression changes. By combining computational and experimental strategies, researchers can effectively translate eQTL findings into meaningful biological insights, advancing our understanding of gene regulation and its impact on human health and disease.

## CONCLUDING REMARKS

In this resource, we provide an introductory guideline for biologists with little or no expertise in bioinformatics who are interested in conducting eQTL analysis. Our guidelines focus on the key steps and software tools for bulk RNA-seq data, which is commonly used for gene expression profiling. However, it is important to note that single-cell RNA sequencing data offer a unique opportunity to investigate cell type–specific gene regulation in complex tissues, providing deeper insights into biological processes and diseases. Recent studies on COVID-19 (Edahiro et al., 2023) and neurological disorders (de Klein et al., 2023) highlighted the power of single-cell eQTL analysis in unraveling cell type–specific regulatory mechanisms underlying disease pathology.

While single-cell approaches provide unprecedented resolution, they also present distinct challenges, such as high technical noise, data sparsity, and the complexity of managing cellular heterogeneity. The large data volumes and the complexity of integrating genetic and transcriptomic information increase computational demands. Additionally, the dynamic nature of single-cell gene expression and the immense burden of multiple testing require careful statistical considerations. Despite these challenges, recent advances in computational tools have improved the integration of single-cell RNA sequencing data into eQTL mapping. Tools such as SCeQTL (Hu et al., 2020), scReQTL (Liu et al., 2021), CellRegMap (Cuomo et al., 2022), and FastGxC (Lu et al., 2021) are specifically designed to address these challenges, enabling more robust and efficient single-cell eQTL analysis.

Furthermore, various other molQTL data types, such as splicing QTLs, methylation QTLs, and chromatin accessibility QTLs, can be utilized to uncover associations between genetic variants and different layers of gene regulation. This approach allows for a more comprehensive understanding of the functional consequences of genetic variation and the identification of key regulatory pathways and networks that can unravel the complex interplay between genetic variation and molecular phenotypes.

## AUTHOR CONTRIBUTIONS

**Byung Su Ko:** Writing – review & editing, Writing – original draft, Conceptualization. **Sung Bae Lee:** Supervision, Conceptualization. **Tae-Kyung Kim:** Writing – review & editing, Supervision, Conceptualization.

## DECLARATION OF COMPETING INTERESTS

The authors declare that they have no known competing financial interests or personal relationships that could have appeared to influence the work reported in this paper.
